# Supplementary material for: Latent instabilities in metallic LaNiO3 films by strain control of Fermi-surface topology
Source: Sci Rep. 2015 Mar 4;5:8746. doi: 10.1038/srep08746 (PMC4348653; doi:10.1038/srep08746)
Supplement: Supplementary Information — Latent instabilities in metallic LaNiO3 films by strain control of Fermi- surface topology [file srep08746-s1.pdf]

Supplementary Information for Latent instabilities in metallic $LaNiO_3$ films by strain control of Fermi-surface topology

Hyang Keun Yoo,[1,2] Seung Ill Hyun,[3] Luca Moreschini,[4] Hyeong-Do Kim,[1,2]* Young Jun Chang,[4,5†] Chang Hee Sohn,[1,2] Da Woon Jeong,[1,2] Soobin Sinn,[1,2] Yong Su Kim,[1,2] Aaron Bostwick,[4] Eli Rotenberg,[4] Ji Hoon Shim,[3,6] and Tae Won Noh[1,2]

[1]*Center for Correlated Electron Systems, Institute for Basic Science (IBS), Seoul 151-747, Republic of Korea*

[2]*Department of Physics and Astronomy, Seoul National University, Seoul 151-747, Republic of Korea*

[3]*Department of Chemistry, Pohang University of Science and Technology, Pohang 790-784, Republic of Korea*

[4]*Advanced Light Source (ALS), E. O. Lawrence Berkeley National Laboratory, Berkeley, California 94720, USA*

[5]*Department of Physics, University of Seoul, Seoul 130-743, Republic of Korea*

[6]*Division of Advanced Nuclear Engineering, Pohang University of Science and Technology, Pohang 790-784, Republic of Korea*

*e-mail: hdkim6612@snu.ac.kr

†e-mail: yjchang@uos.ac.kr

S1. Lattice structures of $LaNiO_3$ films

We monitored surface crystal structures of $LaNiO_3$ (LNO) films using *in situ* reflection high-energy electron diffraction as shown in Figs. S1(a) and S1(b). They show a 1×1 surface crystal structure, and no additional structure is observed. Figures S1(c)–(e) show x-ray diffraction data for thirty-unit cell (UC)-thick LNO films on $LaAlO_3$ (LAO, −1.3% misfit strain) and $SrTiO_3$ (STO, +1.7% misfit strain) substrates. The data were obtained using a high-resolution six-circle x-ray diffractometer at the 9C Beamline of PLS. We are able to obtain *c*-lattice parameters from θ-2θ measurements. All the films show well-defined Kiessig fringes indicating the uniform film thickness and atomically smooth interfaces, surfaces of the $LaNiO_3$ films. Additionally, we confirmed that the LNO films are fully and homogeneously strained over 30 UC, based on the reciprocal space mapping in the (103) plane. Additionally, we calculate the *c*-lattice parameters of LNO films assuming a fixed unit-cell volume for a pseudocubic bulk LNO (a = 3.84 Å). As shown in Fig. S1(f), compared with the calculated values, the measured *c*-lattice parameter in the LNO/LAO exhibits a small decrease and that in the LNO/STO exhibits a large increase. These results indicate that the unit-cell volumes of the LNO films are changed depending on the misfit strain.

To understand this volume change in the strained LNO films, we obtained the lattice structure using the generalized gradient approximation (GGA) calculations. To apply the biaxial strain caused by epitaxial growth on the lattice mismatched substrates, the cubic cell was deformed by −1.7% (+2.0%) along the *a* and *b* directions to account for compressive (tensile) strain, as shown in Figs. S1(g) and S1(h). The system is allowed to relax along the *c* direction to minimize the total energy to within 0.0001 Ry. Distortion of the oxygen octahedron is neglected in the calculations. We found that the results of the GGA calculations show similar volume changes to the experimental data, shown as blue squares in Fig. S1(f). This result indicates that the changes of the unit cell in the strained LNO films may be an

intrinsic property.

S2. Photon-energy scan and k_z resolution

The ten-unit cell-thick LNO film has a three-dimensional band structure similar to that of bulk LNO. Therefore, we needed to have a photon-energy scan to identify appropriate photon energies for the two symmetric planes; the Γ*XM* (*ZRA*) plane was found to be located at slightly different energies of 150 eV (114 eV) for LNO/LAO, 154 eV (117 eV) for LNO on $NdGaO_3$ (NGO), and 157 eV (120 eV) for LNO/STO due to different lattice parameters. Figure S2 shows the details of the photon energy scan. The thick LNO film has a tetragonal structure under the tensile misfit strain. As shown in Fig. S2(a), the following Brillouin zone (BZ) was represented as a schematic diagram with the symbols for high-symmetry momentum points (Γ, *X*, *M*, *Z*, *R*, and *A*). We scanned the photon energy in normal emission, shown as shaded Γ*XRZ* plane in Fig. S2(a). Figure S2(c) showed the k_z scan at the Fermi level (E_F). Based on our calculation of the bulk LNO, we could determine the proper photon energies for the high-symmetry points, such as Γ (157 eV) and *Z* (120 eV) points, of our LNO film. Note that, as shown in Fig. S2(d), the momentum resolution is around 0.13 $Å^{-1}$ along the k_z-direction. It was relatively poor because of the surface scattering and the short mean free path at 100 – 200 eV photon energy [1].

Because ARPES spectra are picked up with a finite k_z resolution Δk_z, any theoretical calculations should consider this effect to compare with ARPES data when the band dispersion is not negligible along the k_z axis. To investigate the Δk_z effect in experiment, we compared the theoretical calculation results without (Fig. S3 i) and with (Fig. S3 ii) Δk_z to the experimental results (Fig. S3 iii). We calculated the Fermi surfaces (FSs) at 0.05, 0.1, 0.15, and 0.2 $Å^{-1}$ away from the Γ and the *Z* points along the k_z-direction. Then, considering the weight in a Lorentzian curve as shown in Fig. S4, we integrated these data to see the Δk_z

effect, to find a much better agreement, especially in the Γ*XM* plane.

We should note that the Δk_z effect is smaller in the *ZRA* plane, compared to that in the Γ*XM* plane. In Figs. S3(b) and (d), there are no big differences in the Δk_z-averaged theoretical calculations except for small broadening. This is attributed to the small change of band structure along the k_z-direction near the *ZRA* plane as shown in Figs. 3(e) and (f). This is also revealed in the experimental result. We obtained the momentum distribution curves (MDCs) along the *X*Γ*X*, the *MXM* and the *ARA* lines in *t*-LNO as shown in Fig. S5. The line profiles along the *X*Γ*X* and the *MXM* lines are broad and have some complicated structure as seen in Figs. S5(a) and (b), which can be understood by the Δk_z effect. On the other hand, the MDC along the *ARA* line is fitted with a single Lorentzian curve, as shown in Fig. S5(c). The full width at half maximum is around 0.08 $Å^{-1}$, which indicates that the scattering lenght is around 25 Å. We note that the reported scattering length of bulk-like LNO under tensile strain is 12 Å [2], which guarantees much better quality of our thin films.

S3. Misfit strain induced changes of the electronic structure in metallic LNO films.

We can explain the misfit strain-induced changes in the band dispersions of the LNO films by considering e_g-orbital splitting and changes in the bandwidth *W*. The two e_g orbitals, d_{3z2-r2} and d_{x2-y2}, are degenerate in a pseudocubic bulk LNO [3]; however, the contraction or elongation of the equatorial Ni–O bond length, d_{Ni-O}, due to the misfit strain splits the degenerate e_g orbital levels. For example, under compressive strain, the energy level of the d_{3z2-r2} (d_{x2-y2}) orbital decreases (increases) due to a decreasing (increasing) hybridization with the surrounding oxygen ions [4]. The *W* of the e_g orbitals can also change in response to changes in d_{Ni-O} [4]. For instance, under compressive strain, hybridization with apical oxygen ions in the NiO_6 octahedra decreases due to an elongation of the Ni–O bond length along the

z-direction. Therefore, the W corresponding to the d_{3z2-r2} orbital is decreased under compressive strain, even though the energy level of that becomes lower.

As shown in Fig. S6, using the dynamical mean-field theory with the GGA (GGA+DMFT) calculations, we obtained the e_g-orbital characters in the band dispersions along the $M\Gamma XMAZRA$ line. The d_{x2-y2} and d_{3z2-r2} orbitals are shown by blue- and red-scale images, respectively. In Figs. S6(a) and S6(b), we can see that, below the Fermi-energy, the electron band at the Γ point and the hole band centered at the A point have a dominant d_{x2-y2} orbital character. On the other hand, the band along $M\Gamma XM$ shows a dominant d_{3z2-r2} orbital character, as shown in Fig. S6(c) and S6(d). Therefore, with these results of calculations, we can understand the downward (upward) shift of the band structure along the $AZRA$ ($M\Gamma XM$) line as the strain states change from compressive to tensile (see Figs. 1 and 2). Note that the Fermi-surface topology change is accompanied with this energy level shift. Figure S7 shows the Fermi-surface topology change more clearly.

However, although the electron band centered at the Γ point has a dominant d_{x2-y2} orbital character, the band minimum exhibits a slight upward shift with tensile strain. To understand this behavior, the strain-induced change in the W should be also considered. As shown in Fig. S6, we can clearly observe that the W of the d_{x2-y2} orbital decreases with tensile strain, although its energy level becomes lower. This indicates that the band minimum at the Γ point may become higher in energy due to the decrease in the W of the d_{x2-y2} orbital with tensile strain.

We note that the LNO film remains a metallic phase irrespective of misfit strain. There is a result to claim that LNO film might have a gap structure under tensile strain [5]. However, as shown in Fig. S8, we found that the LNO films are metallic, irrespective of misfit strains. Especially, as shown in Fig. S8(a),(b)-v,vi, the energy-distribution-curves (EDCs) are symmetrized at the E_F-crossing points α and β, indicated by red arrows. All the symmetrized

EDCs show the peak structure at the E_F, which implies the metallic phases of LNO films, not the insulating gap structure.

S4. Fermi-surface superstructures in LNO films.

A careful examination of the FS map in Fig. 3(c) and 3(d) reveals faint lines, implying an existence of superstructure in the FS. First of all, to clarify the superstructure with **Q** = (1/2,1/2,1/2) modulation, we constructed the constant-energy-surface (CES) map of the LNO film on NGO substrate in the Γ*XM* plane at 30 meV using a log scale, as shown in Fig. S9(a). However, the signal of the FS superstructure in the Γ*XM* plane is too weak to notice. Thus, to visualize the FS superstructure, we obtained the MDC along the dashed line in Fig. S9(a). As shown in Fig. S9(b), the MDC shows additional peaks indicated by black arrows. The peak structure and the peak separation are nearly the same with the hole pocket centered at the A point. This result supports **Q** = (1/2,1/2,1/2) modulation in FS. Note that, Fig. S10(a) shows a CES map in the *ZRA* plane of LNO/NGO at a binding energy of 30 meV using a log scale. And figure S10(b) reveals the schematic CES maps after whole BZ folding with **Q** = (1/2,1/2,1/2). This result indicates that the LNO/NGO has the same FS superstructure with LNO/LAO, as shown in Fig. 4(a).

To investigate the superstructure of the tensile-strained LNO film on STO substrate more clearly, we constructed CES maps in the Γ*XM* and the *ZRA* planes at a binding energy of 30 meV using a log scale, as shown in Figs. S11(a) and S11(b). Signal of the FS superstructure in the *ZRA* plane is quite clear, but that in the Γ*XM* plane is too weak to notice. Thus, to visualize the FS superstructures, we obtained the MDCs along α and β cuts in the Γ*XM* and the *ZRA* planes, respectively, as indicated by dashed lines in Figs. S11(a) and S11(b). The MDCs in the Γ*XM* and the *ZRA* planes show the peak structures and the peak separations are nearly the same as shown in Fig. S11(c). The superstructure along the β cut is considered as a

replica of the hole pocket at the *A* point, so that along α cut can originate from the same FS. This result indicates that the FS superstructure in the tensile-strained LNO film should have a periodic modulation with $\mathbf{Q} = (1/4,1/4,1/4)$ for the hole pockets. Note that it cannot be reproduced by a periodic modulation with $\mathbf{Q} = (1/4,1/4,0)$, which rules out the possibility of a $2\sqrt{2} \times 2\sqrt{2}$ surface reconstruction.

S5. Details of calculation methods.

Calculations of the pseudo-cubic and the strained LNO bulk were performed using a charge-self-consistent DMFT and a full-potential GGA implemented in WIEN2k [6-8]. We considered the effects of in-plane strain via a contraction or an elongation of the $d_{\mathrm{Ni-O}}$ in the NiO_6 octahedra. The GGA+DMFT calculations performed with −1.7% strain which had the following lattice constants: a = 3.78 Å and c = 3.88 Å; with +2.0% strain, the lattice constants were a = 3.916 Å and c = 3.798 Å, which shows a consistent behavior with the results of x-ray diffraction measurements. The lattice parameters were optimized with a tolerance of 0.0001 Ry in the GGA calculation. The Perdew–Burke–Ernzerhof parameterization of the GGA (PBE-GGA) [9] was used to treat the exchange correlation because it was able to reproduce pseudo-cubic lattice parameters that were consistent with the experimental values.

In the GGA+DMFT calculations, the local self-energy $\Sigma(\omega)$ of Ni 3*d* electrons was calculated by solving the corresponding quantum-impurity problem using the continuous-time quantum-Monte-Carlo method at a temperature of 230 K (50 eV^{-1}) [10]. The value of the Hubbard U was 14.0 eV, and the Hund's coupling parameter was J = 0.7 eV. Note that the value of U exceeds the conventional value for the rare-earth nickelate system [11-13], because a dynamic screening by uncorrelated bands is considered within an energy range of [−10 eV, +10 eV] centered on the Fermi energy.

In order to check if any density-wave instabilities exist, Lindhard response functions for the LNO films were calculated. The Lindhard response function [14,15] will show a logarithmic divergence at the q vector corresponding to a wave-vector for charge- or spin-density wave instabilities [16-18]. Note that, in the description of the Fermi surface, the GGA+DMFT quasiparticle spectrum shows little deviation from the GGA results. So, we used the GGA eigenvalues for the calculation of the Lindhard response function. The Lindhard response function was calculated from the GGA eigenvalues of random *k*-points generated by a linear interpolation of a $100 \times 100 \times 100$ *k*-point grid in the full Brillouin zone, including the Γ point. As the GGA+DMFT quasi-particle spectrum is well defined and shows a little deviation from the GGA results at the Fermi energy, GGA eigenvalues were used for the calculation of the Lindhard response function. As shown in Fig. S12, the susceptibility of LNO with tensile strain has a sharper peak around $\mathbf{Q}$ = (1/4,1/4,1/4), compared with that for the compressive strain. Note that the susceptibility, $\chi(q)$, are normalized by the value of the $\chi(0)$.

References

[1] V. N. Strocov, M. Shi, M. Kobayashi, C. Monney, X. Wang, J. Krempasky, T. Schmitt, L. Patthey, H. Berger, and P. Blaha, Phys. Rev. Lett. **109**, 086401 (2012).

[2] A. V. Boris, Y. Matiks, E. Benckiser, A. Frano, P. Popovich, V. Hinkov, P. Wochner, M. Castro-Colin, E. Detemple, V. K. Malik, C. Bernhard, T. Prokscha, A. Suter, Z. Salman, E. Morenzoni, G. Cristiani, H.-U. Habermeier, B. Keimer, Science **332**, 937 (2011).

[3] N. Hamada, J. Phys. Chem. Solids **54**, 1157-1160 (1993).

[4] Y. Tokura, and N. Nagaosa, Science **288**, 462-468 (2001).

[5] J. Chakhalian, J. M. Rondinelli, Jian Liu, B. A. Gray, M. Kareev, E. J. Moon, N. Prasai, J. L. Cohn, M. Varela, I. C. Tung, M. J. Bedzyk, S. G. Altendorf, F. Strigari, B. Dabrowski, L. H. Tjeng, P. J. Ryan, and J.W. Freeland, Phys. Rev. Lett. **107**, 116805 (2011).

[6] G. Kotliar, S. Savrasov, K. Haule, V. Oudovenko, O. Parcollet, and C. Marianetti, Rev. Mod. Phys. **78**, 865–951 (2006).

[7] K. Haule, C.-H. Yee, and K. Kim, Phys. Rev. B **81**, 195107 (2010).

[8] P. Blaha, K. Schwarz, G. K. H. Madsen, D. Kvasnicka, and J. Luitz, WIEN2k (Vienna University of Technology, Vienna, Austria, 2001).

[9] J. P. Perdew, K. Burke, and M. Ernzerhof, Phys. Rev. Lett. **77**, 3865 (1996).

[10] K. Haule, Phys. Rev. B **75**, 155113 (2007).

[11] X. Deng, M. Ferrero, J. Mravlje, M. Aichhorn, and A. Georges, Phys. Rev. B **85**, 125137 (2012).

[12] P. Hansmann, A. Toschi, X. Yang, O. Andersen, and K. Held, Phys. Rev. B **82**, 235123 (2010).

[13] B. Lau and A. J. Millis, Phys. Rev. Lett. **110**, 126404 (2013).

[14] J. Lindhard, Kgl. Danske Videnskab. Selskab Mat.-Fys. Medd. 8, (1954).

[15] N. W. Ashcroft and N. D. Mermin, Solid State Physics (Thomson Learning, Toronto,

1976).

[16] E. Fawcett, Rev. Mod. Phys. **60**, 209–283 (1988).

[17] G. Grüner, Rev. Mod. Phys. **60**, 1129–1181 (1988).

[18] A. Overhauser, Phys. Rev. **128**, 1437–1452 (1962).

Figure Captions

FIG. S1. (a), (b) Reflection high-energy electron diffraction patterns for $LaNiO_3$ (LNO) films on $LaAlO_3$ and $SrTiO_3$ substrates. (c)–(e) θ-2θ measurements and reciprocal space mapping in the (103) plane. (f) Measured *c*-lattice parameters for LNO films from the θ-2θ measurements are different from the calculated values assuming a fixed unit cell volume for a bulk LNO. (g,h) The generalized gradient approximation (GGA) calculations showed similar changes of the *c*-lattice parameters to the measured data.

FIG S2. (a) The Brillouin zone (BZ) of the tetragonal structure for thick LNO film under tensile misfit strain. The symbols for high-symmetry momentum points (Γ, *X*, *M*, *Z*, *R*, and *A*) come from the BZ of the tetragonal structure. (b) Range of photon-energy scan projected to Γ*XRZ* planes of the BZ. Dashed circle is the predicted circular band centered at the Γ point. (c) Photon-energy scan of ten-unit cell-thick LNO film, which shows the circular band around the Γ point. (d) Momentum-distribution-curve (MDC) along blue arrow in (c). The MDC is fitted by two-Lorentzian curves, which shows that the k_z resolution is around 0.13 $Å^{-1}$.

FIG S3. Fermi surfaces (FSs) of (a),(b) compressive-strained LNO (*c*-LNO) and (c),(d) tensile-strained LNO (*t*-LNO) in the Γ*XM* and the *ZRA* planes. i and ii indicate the dynamical mean-field theory combined with the GGA (GGA+DMFT) results without and with k_z-average, respectively, whereas iii is an experimental results.

FIG. S4. Lorentzian curve with 0.13 full width at half maximum (red curve). The blue area indicates the k_z averaged region in the calculation.

FIG. S5. MDCs along the $X\Gamma X$, the *MXM* and the *ARA* lines in *t*-LNO film. The line profiles in (a) and (b) show the broad feature, which implies significant k_z-average effect. The MDC in (c) shows sharp peak feature and it is fitted by single Lorentzian curve.

FIG S6. The e_g-orbital, d_{3z2-r2} and d_{x2-y2}, characters in the band dispersions of LNO films with (a,c) −1.7% *c*-LNO and (b,d) +2.0% *t*-LNO, obtained from the GGA+DMFT calculations.

FIG S7. FS maps and MDC stacking plots in the ΓXM and the *ZRA* planes of (a,c) *c*-LNO and (b,d) *t*-LNO, respectively.

FIG S8. Band dispersions and energy-distribution-curve (EDC) stacking plots along the ΓX and the *RA* lines of (a) *c*-LNO and (b) *t*-LNO, respectively. As shown in (a),(b)-v,vi, the EDCs are symmetrized at the Fermi level (E_F)-crossing points α and β, indicated by red arrows.

FIG S9. (a) Constant-energy-surface (CES) map of the LNO film on $NdGaO_3$ substrate at 30 meV in the ΓXM, using a log scale. (b) MDC along the gray dashed line in (a). The MDC shows additional peaks indicated by black arrows, which supports **Q** = (1/2,1/2,1/2) modulation in the Fermi surface.

FIG S10. (a) Experimental CES maps in the ZRA plane at 30 meV of LNO film on $NdGaO_3$ substrate using a log scale. (b) Schematic CES maps after folding the BZ with **Q** = (1/2,1/2,1/2).

FIG S11. CES maps at 30 meV of *t*-LNO film in (a) the Γ*XM* and (b) the *ZRA* planes, using a log scale. (c) Comparison of MDCs along α and β cuts in the Γ*XM* and the *ZRA* planes, respectively.

FIG S12. Susceptibilities of the LNO films under two different strain states calculated from the GGA results. The tensile strain state has a sharper peak around **Q** = (1/4,1/4,1/4) than the compressive strain state, as indicated by an arrow.

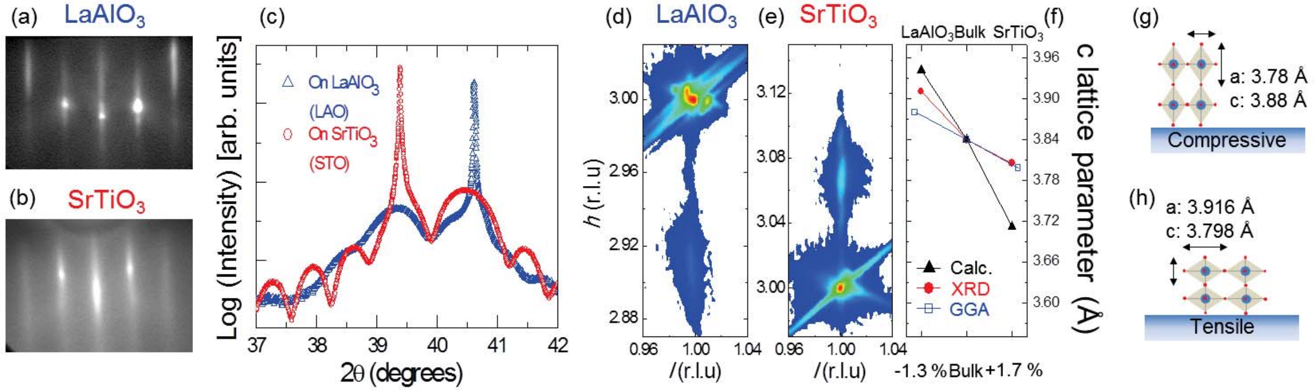

Supplementary Figure 1 (H. K. Yoo *et al*.)

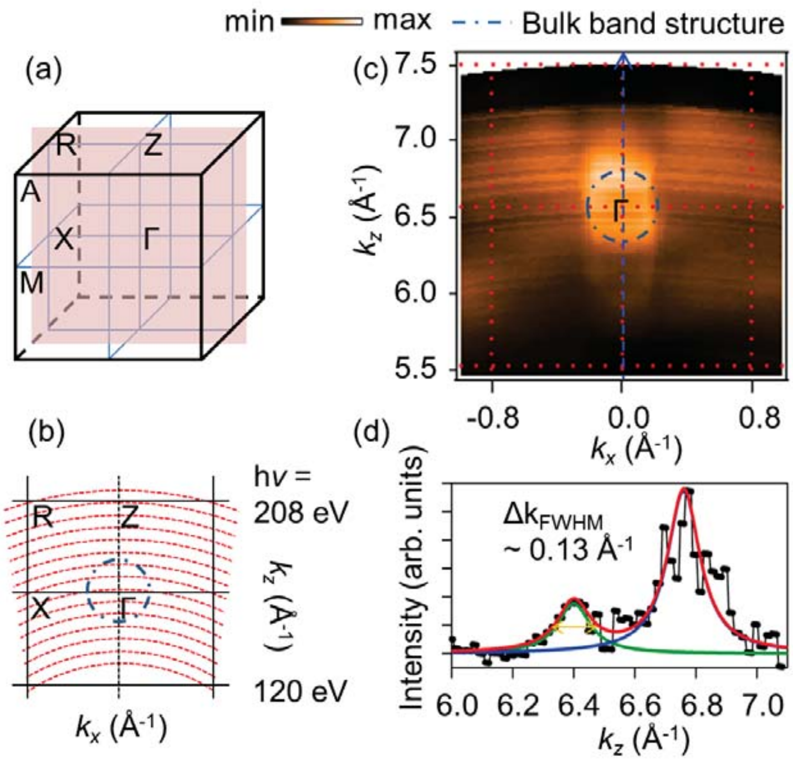

Supplementary Figure 2 (H. K. Yoo *et al*.)

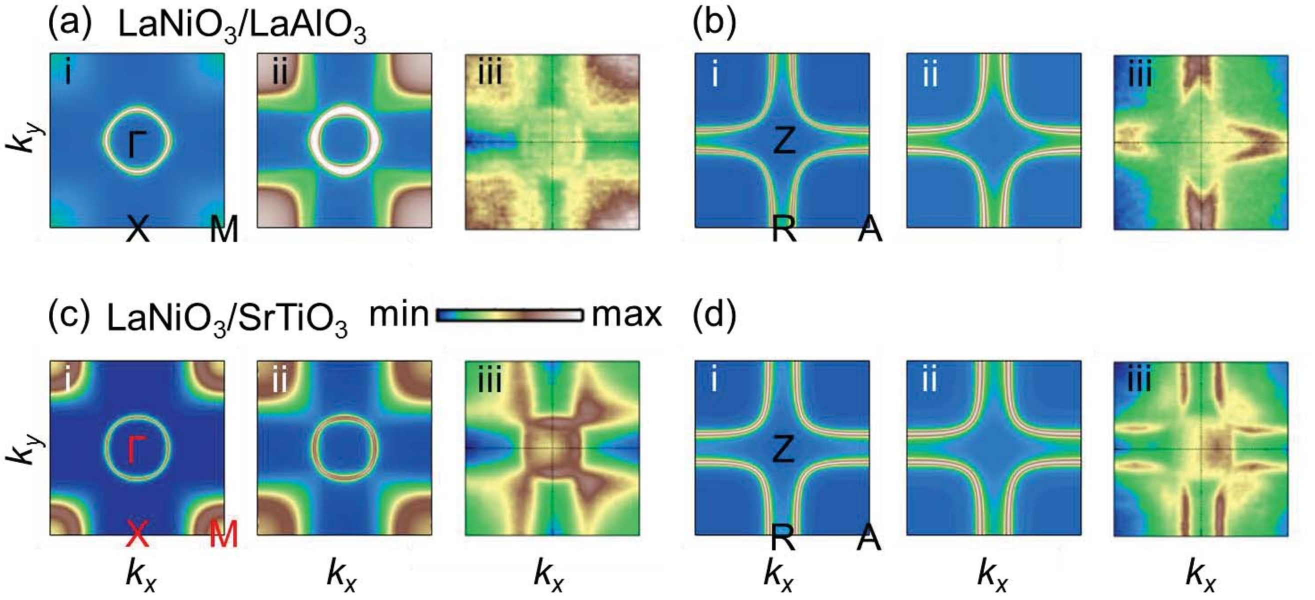

Supplementary Figure 3 (H. K. Yoo *et al*.)

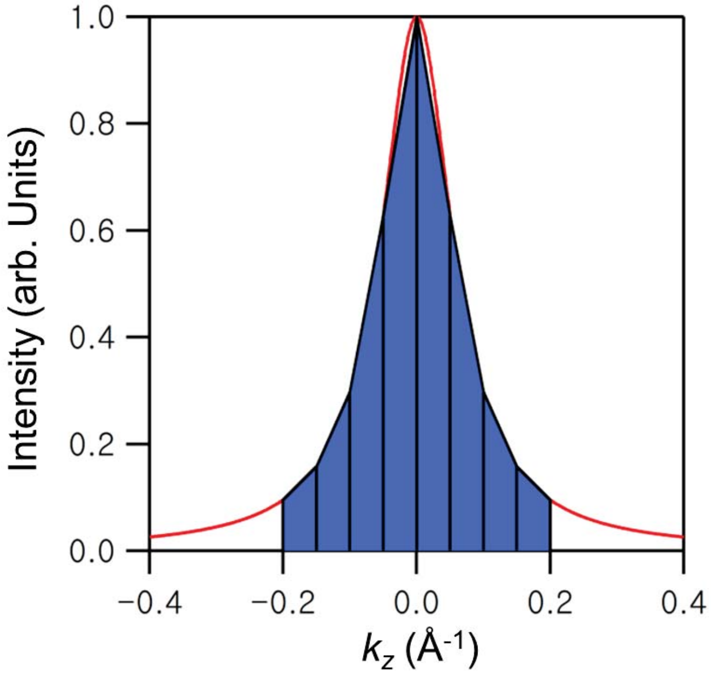

Supplementary Figure 4 (H. K. Yoo *et al*.)

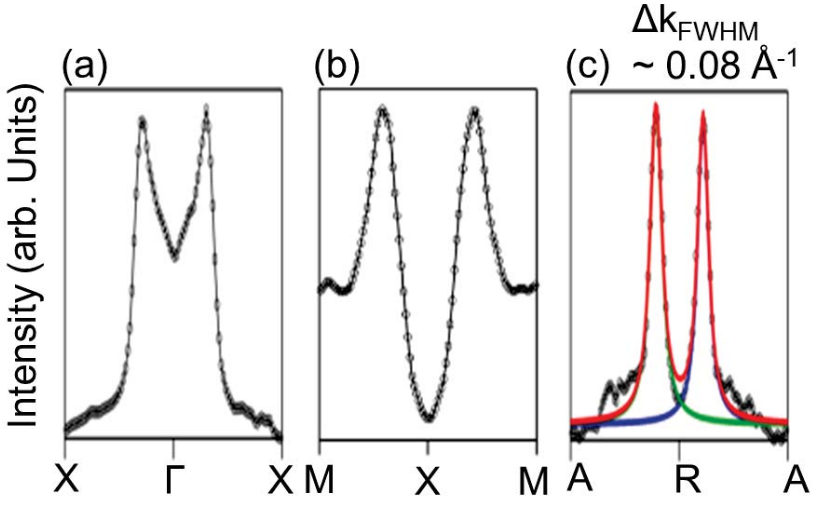

Supplementary Figure 5 (H. K. Yoo *et al*.)

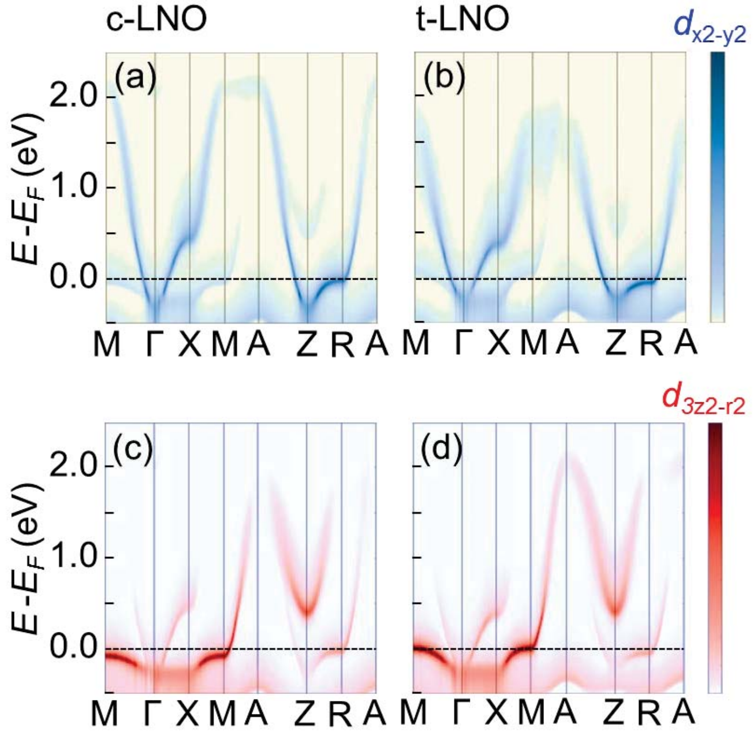

Supplementary Figure 6 (H. K. Yoo *et al*.)

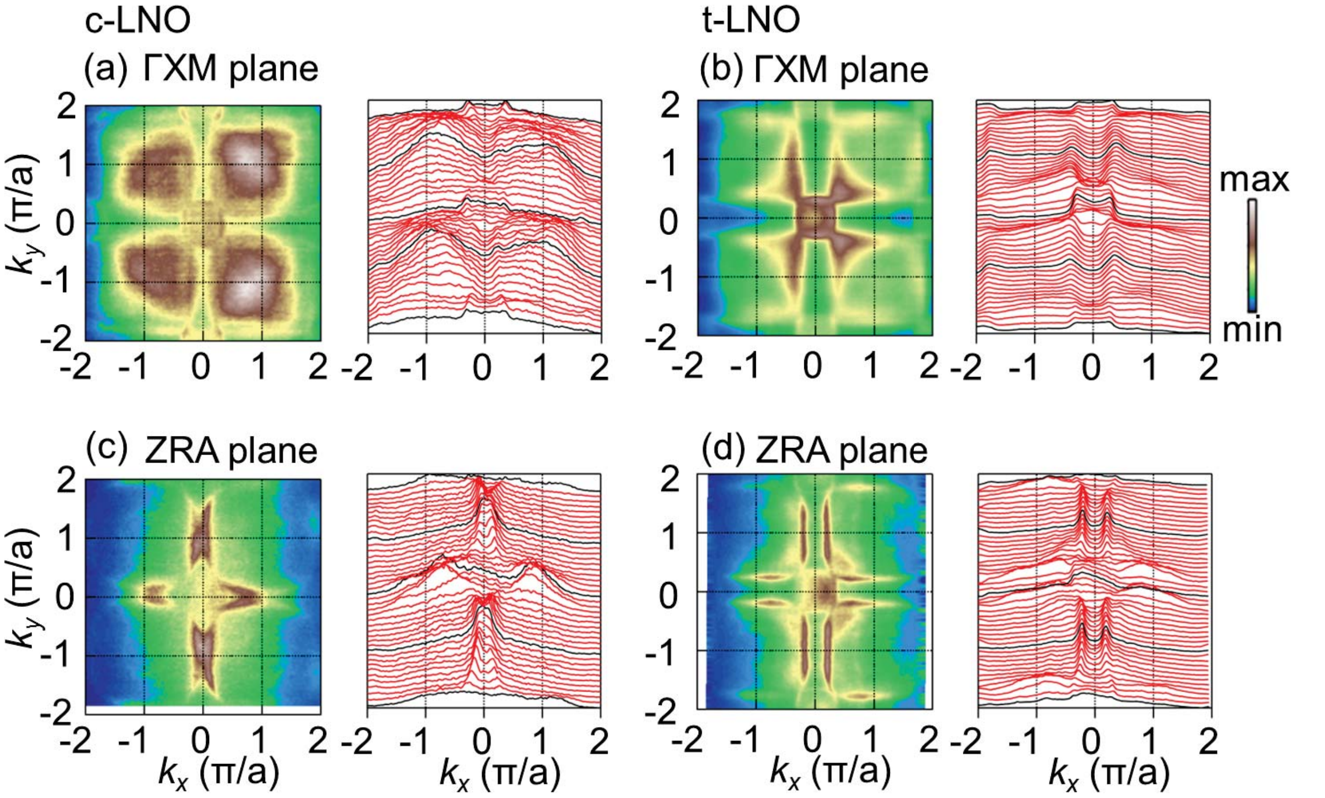

Supplementary Figure 7 (H. K. Yoo *et al*.)

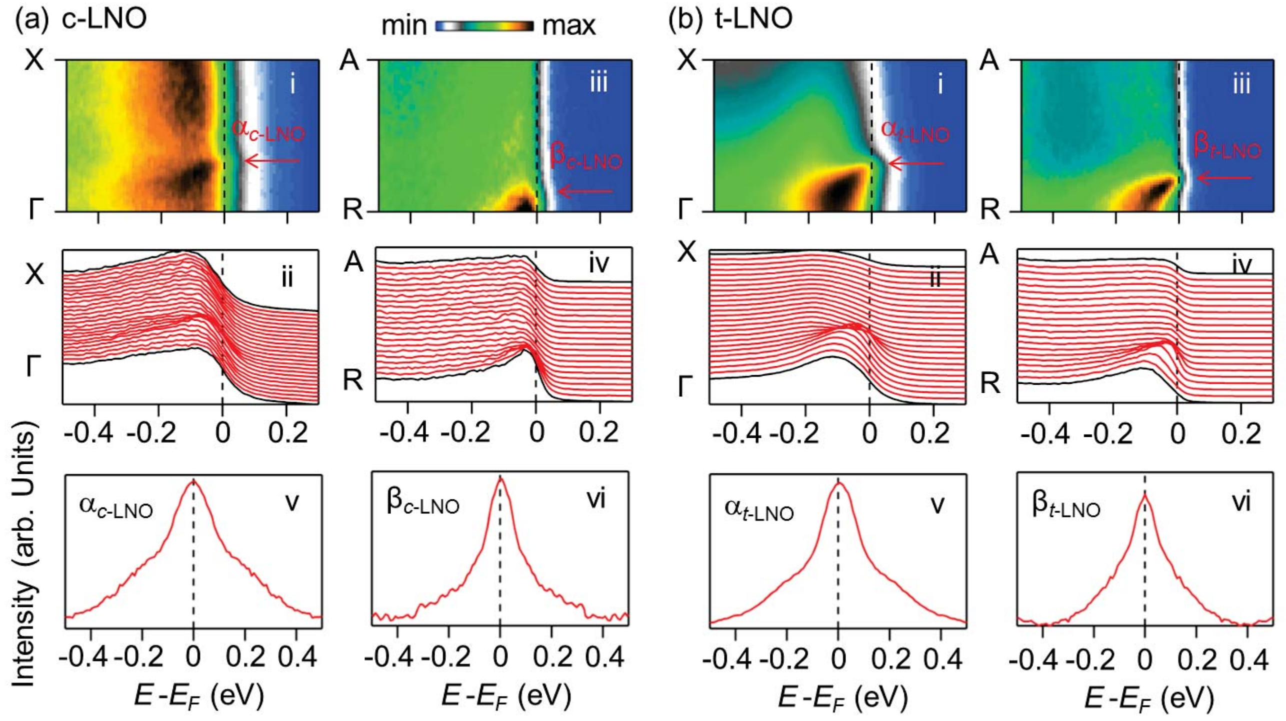

Supplementary Figure 8 (H. K. Yoo *et al*.)

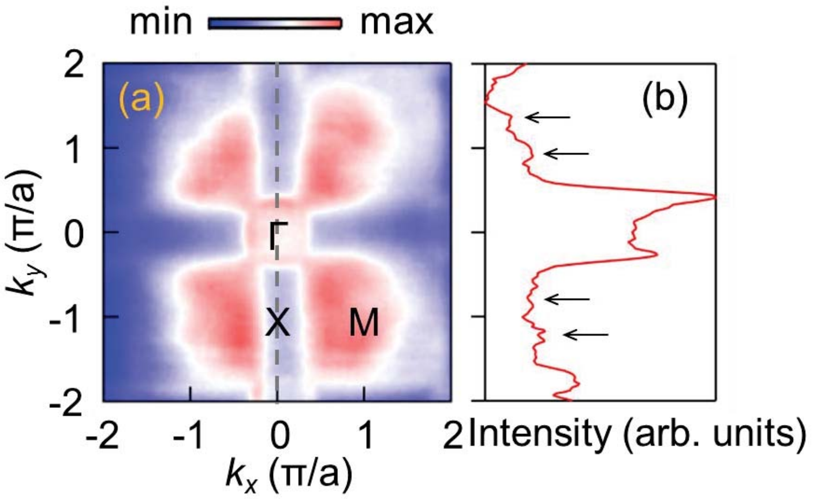

Supplementary Figure 9 (H. K. Yoo *et al*.)

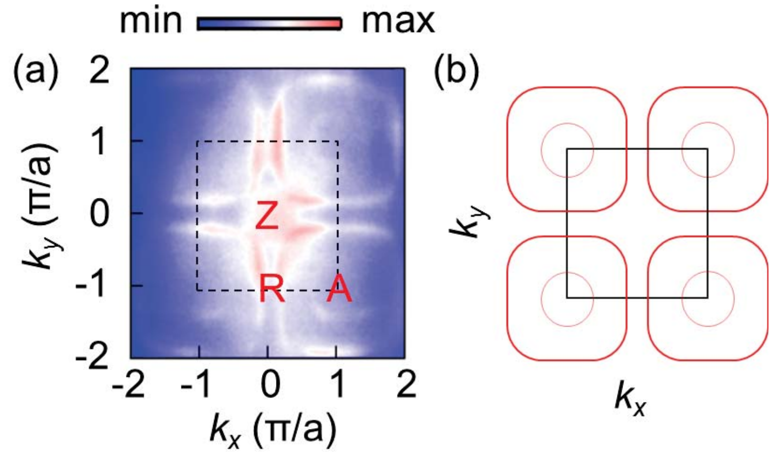

Supplementary Figure 10 (H. K. Yoo *et al*.)

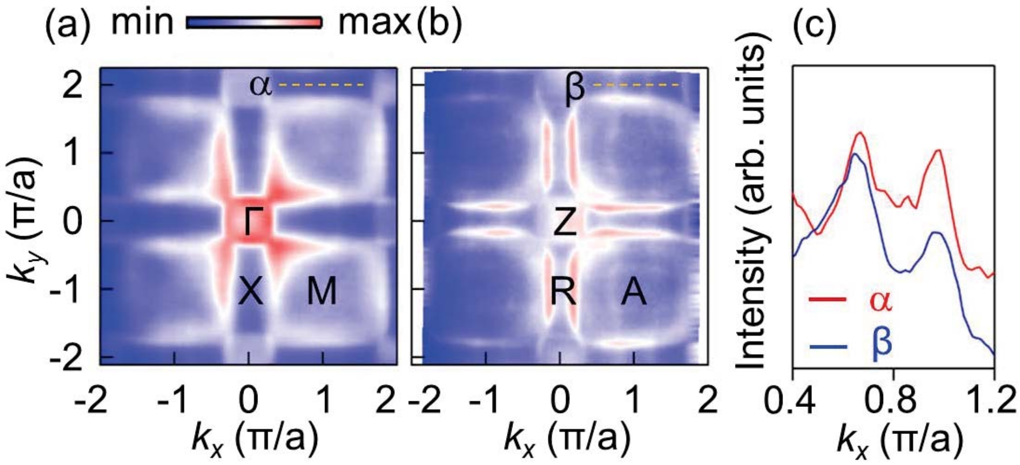

Supplementary Figure 11 (H. K. Yoo *et al*.)

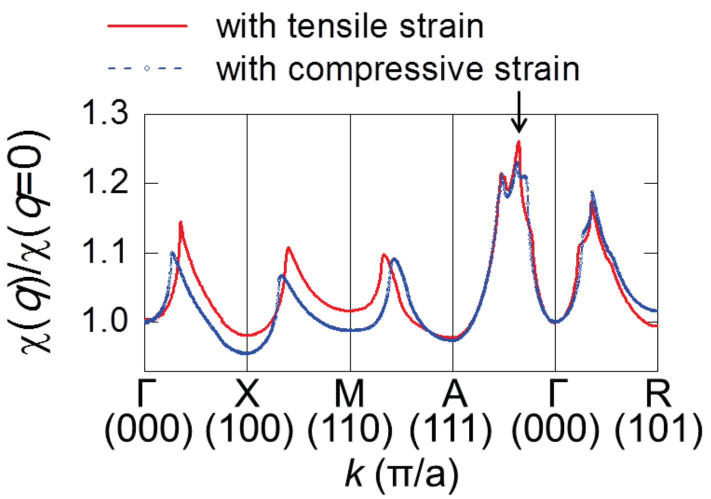

Supplementary Figure 12 (H. K. Yoo *et al*.)
